# Situationally-Sensitive Knowledge Translation and Relational Decision Making in Hyperacute Stroke: A Qualitative Study

**DOI:** 10.1371/journal.pone.0037066

**Published:** 2012-06-01

**Authors:** Madeleine J. Murtagh, Duika L. Burges Watson, K. Neil Jenkings, Mabel L. S. Lie, Joan E. Mackintosh, Gary A. Ford, Richard G. Thomson

**Affiliations:** 1 Institute of Health & Society, Newcastle University, Newcastle upon Tyne, United Kingdom; 2 Department of Health Sciences, College of Medicine, Biological Sciences and Psychology, University of Leicester, Leicester, United Kingdom; 3 Centre for Translational Research, Wolfson Research Institute, Durham University, Stockton, United Kingdom; 4 Geography, Politics and Sociology, Newcastle University, Newcastle upon Tyne, United Kingdom; 5 Institute for Ageing & Health, Faculty of Medical Sciences, Newcastle University, Newcastle upon Tyne, United Kingdom; 6 Newcastle upon Tyne Hospitals NHS Foundation Trust, Newcastle upon Tyne, United Kingdom; 7 Institute of Cellular Medicine, Faculty of Medical Sciences, Newcastle University, Newcastle upon Tyne, United Kingdom; Charité Universitaetsmedizin Berlin, Germany

## Abstract

Stroke is a leading cause of disability. Early treatment of acute ischaemic stroke with rtPA reduces the risk of longer term dependency but carries an increased risk of causing immediate bleeding complications. To understand the challenges of knowledge translation and decision making about treatment with rtPA in hyperacute stroke and hence to inform development of appropriate decision support we interviewed patients, their family and health professionals. The emergency setting and the symptomatic effects of hyper-acute stroke shaped the form, content and manner of knowledge translation to support decision making. Decision making about rtPA in hyperacute stroke presented three conundrums for patients, family and clinicians. 1) How to allow time for reflection in a severely time-limited setting. 2) How to facilitate knowledge translation regarding important treatment risks and benefits when patient and family capacity is blunted by the effects and shock of stroke. 3) How to ensure patient and family views are taken into account when the situation produces reliance on the expertise of clinicians. Strategies adopted to meet these conundrums were fourfold: face to face communication; shaping decisions; incremental provision of information; and communication tailored to the individual patient. Relational forms of interaction were understood to engender trust and allay anxiety. *Shaping decisions* with patients was understood as an expression of confidence by clinicians that helped alleviate anxiety and offered hope and reassurance to patients and their family experiencing the shock of the stroke event. Neutral presentations of information and treatment options promoted uncertainty and contributed to anxiety. ‘Drip feeding’ information created moments for reflection: clinicians literally *made* time. *Tailoring information* to the particular patient and family situation allowed clinicians to account for social and emotional contexts. The principal responses to the challenges of decision making about rtPA in hyperacute stroke were *relational* decision support and *situationally-sensitive* knowledge translation.

## Introduction

Stroke is a leading cause of mortality and severe disability in the UK [1]. Effective implementation of the only proven immediate treatment for acute ischaemic stroke - thrombolysis with recombinant tissue plasminogen activator (rtPA) - could lead to more than 1500 patients per annum fully recovering from their stroke who would otherwise have been left with long term disability [1]. RtPA must be given within four and a half hours from onset of symptoms and treatment is more effective the earlier it is given within this time window [2]. There are, however, risks of early symptomatic intracranial haemorrhage, in 2–3% of patients, two thirds of which are fatal. Guidelines recommend a “door to needle time” of 30 minutes, leaving little time for patient and family decision making. Decision making, therefore, occurs in an emergency context and may involve complex issues that include the consideration of the evidence for overall benefits of therapy alongside risks of worse outcome (or death) from the adverse effects of the treatment [3], [4]. Knowledge translation and decision making in the emergency setting presents unique challenges [5].

Within health care there is now an expectation that the biomedical evidence upon which medical treatment protocols are based is communicated to patients and their families; that the science be translated to the bedside [6], [7]. This knowledge translation process is also part of expectations that health professionals should pay due respect to patients and in doing so promote their autonomy [6], [8]. Shared decision making (SDM) is one strategy of knowledge translation which aims to promote ethical practice. Within the field of shared decision making, research and intervention development (largely in the form of patient decision aids) has sought to establish how patient knowledge might be enhanced and their values and preferences might be incorporated into treatment decisions[9]–[11]. This work has demonstrated that the desire for involvement in decision making varies dependent upon a number of contextual factors [12] and may include gender, education, socio-economic position and age [13]. Some studies have shown that severity of disease shapes preference for involvement in decision making [13]. The range of metapreferences for involvement (that is the preference for particular processes of decision making not simply the preference for a particular choice between one or more outcomes [14]) mean that we cannot assume that involving patients in decision making about a complex intervention is *always* appropriate, or appropriate in *all* contexts or circumstances any more than the paternalistic medicine, to which SDM is responding, might once have assumed that it was *rarely* appropriate to involve patients in decisions about their care. Davies and Elwyn argue that a ‘mandatory autonomy’, that is an *expectation* of involvement in decision making, is inappropriate [15]. Despite the apparent contravention of conventional bioethics it is clear, from studies of patients’ perspectives, shared decision making in practice and metapreferences, that appropriate knowledge translation is complex and ethical practice doesn’t necessarily imply autonomous decision making as traditionally understood. In healthcare settings there are a number of circumstances which may be *apparently* autonomy threatening and where such phenomena can be investigated. Decision making about thrombolysis for stroke, in the emergency setting where time pressures intensify the demands on decision making and for which the effects of the stroke itself may compromise decision making, is one such example.

Taking the complexity of the decision making context into account, we examined, in interviews with patients, family and clinicians, knowledge translation and engagement of patients and their families in decision making about rtPA. Our objective was to elicit the perspectives of these key participants in the emergency treatment trajectory and to thereby understand, from their perspective, the challenges of knowledge translation and decision making about treatment with rtPA in hyperacute stroke and so to inform development of appropriate decision support.

## Methods

### Ethics Statement

The study was granted ethical approval by the Northumberland Research Ethics Committee and Research Governance approval from participating Hospital Trusts. Written informed consent was sought from all participants prior to participation. Participants were specifically asked to consent to the recording and transcribing of face to face interviews and assured of their confidentiality and anonymity.

### Design, Data Collection and Analysis

We conducted semi-structured interviews on decision making about rtPA in the hyperacute setting with participants from three stroke units (identified as A, B and C below) in north east England: 1) patients with a recent stroke (7+/−2 days after stroke), irrespective of treatment received; 2) families (broadly defined) of such patients; and, 3) health professionals involved in decision making and knowledge translation (information provision) about thrombolysis. In qualitative research the sample is selected purposively (i.e. to include those with relevant expertise or experience – here clinicians, patients and their family) and data collected until thematic or theoretical saturation (i.e. the point at which no new themes or ideas are forthcoming). Therefore the sample is representative in that it accurately reflects the range of views available in the relevant population (clinicians, patients and family involved in emergency stroke). However it does not tell us the proportions of participants in the wider population of patients, family members or clinicians who hold these views.

Patients were identified by health care staff in each unit. The patients and their families were given information about the study which included contact details for the research associates and research leads (MM and RGT). Patients were interviewed if they had capacity for engagement in decision-making at the time of the decision and interview, as determined by the responsible stroke specialist. Clinical participants were recruited via direct contact from the research associates. At the time of the interview the study was explained verbally and in writing and participants’ consent was obtained in writing.

The semi-structured interviews followed a question guide (shown below) which covered participants’ perspectives and experiences on: engagement in health care decision making and information provision in general, and about rtPA in particular; the availability, appropriateness and preferences for information provision, including type and format; and factors important to decisions about rtPA in the emergency setting. Interviewers covered topics in the question guide but allowed sufficient flexibility for participants to raise issues that were important to them and that were not necessarily detailed in the question guide. Interviews included individual, paired and conjoint interviews to respect participants’ wishes; in four the patient and family members were interviewed together; in one, two clinical staff were interviewed together. Interviews were undertaken in a place of the participant’s choosing (mostly in a quiet room close to the ward) and lasted between 20 minutes and one hour.

Question Guide: Patients/carers

Tell me what happened immediately after you (or the person you were caring for) was/were taken into medical care for stroke?

Were you given any information about available immediate treatments?

How do you/would you feel about being involved in decision making about a treatment that needs to be given urgently when you (or a relative) have just had a stroke?

For participants who were involved in decision making about rtPA:

What was most important to you in making a decision about thrombolysis immediately after being admitted with a stroke?

What sort of information would you like to have at a time when a quick decision needs to be made about early treatment? What other support do you feel would be helpful?

How do/would you feel about a risk of having a stroke as a result of the treatment?

Is there anything else that you think might be important to helping people make a decision about thrombolysis?

Question Guide: Clinical Staff

How do you feel about patient engagement in decision making?

What does shared decision making mean to you?

What are the benefits and challenges of involving patients and carers in decision making and consent?

What is your experience of patient and clinician involvement in decision making in relation to hyper-acute stroke?

What information is available for patients and carers for hyper-acute stroke and thrombolytic treatment? Do you think this is appropriate/useful?

What would you rather see was available at this time? What form of information or decision support do you think would be useful to you or to patients/carers?

What factors do you think need to be considered when making a decision about thrombolysis?

What factors do you think are important to support effective consent?

How would decision making work in relation to your ideal model(s)?

Interviews were conducted by experienced qualitative research associates employed by Newcastle University (DLBW, MLSL, JEM). None of the interviewers had involvement in the care of the patients. All interviews were audio-recorded and transcribed. The text of these transcripts formed the data for analysis. Thematic analysis was undertaken. Thematic analysis firstly involves deep: familiarisation with the texts. This was achieved by each researchers (DLBW, MLSL, KNJ, JEM, MJM, RT) reading and rereading the transcripts (often in conjunction with listening to the audio recordings) and, through regular meetings, discussing recurrent themes and patterns and emergent propositions about the interpretation of the data. The texts were coded (by MLSL) based on themes and sub-themes. Discourse analysis [16], across the themes and sub-themes, of patterns and rationales within participants’ accounts of decision making was then undertaken (by MM) to elucidate the parameters of appropriate and acceptable decision support in this emergency setting. In presenting this analysis we include representative extracts chosen for inclusion because they best represent the finding under discussion. Where discussion in the interviews was directed specifically to clinicians (e.g. in relation to their clinical practice), this is reflected in the preponderance of clinicians’ quotations. In cases where patients and their family were, by their choice, interviewed together the extracts form part of group discussion. As these discussions can be very long the most relevant part of the quotation was extracted for inclusion. These quotations therefore may or may not include all patient or family members. Interrogation of the validity of interpretations was undertaken in three ways: first, subjecting the interpretations of the qualitative analysis to challenge by clinical and epidemiological researchers (GAF and other clinicians in the broader research team); second, deliberative identification of negative cases [17]; and, third by presenting the reader with sufficient representative quotations that they themselves may judge the coherence of the interpretations (Potter, 1996). Representative quotes are anonymised with explanatory text and non-verbal activity in square brackets.

## Results

Sixty two participants took part in 58 semi-structured interviews. Twenty three interviews were held with clinicians, 35 interviews included patients (22) and family members (15).

### Decision Making in the Emergency Setting

#### You have to make an instant decision

Time constraints and patient capacity, in the context of the availability of a treatment with both risks and benefits, shaped the processes and practices of knowledge translation and decision making in this setting.

*You have to make an instant decision, almost. (Family(Fam)B04)*

*It is an emergency situation and you are made aware very much so of what an emergency it is and you have to make that decision now. (FamB07)*

*[rtPA] is effective, but there are potentially lethal, you know, lethal complications. In such, such occasions it becomes all the more difficult, less time for the patient to make the decision and less time for the clinician to make the decision so we have to really work hard in those three hours. Time is the crucial [factor] in this scenario. The earlier the better, you cannot be losing on the time (Doctor(Doc)C03)*

Often there were only a few minutes for the clinical team to communicate information to a patient and their family about treatment, to discuss the pros and cons, then make a final decision and implement treatment.

*We got there erm, and walked into a cubicle where [the patient] was and before I even had a chance to give him a cuddle or a kiss or anything it was “we’ve got to get on this it’s a quick”. [we are told] “we’ve got 10 minutes to sort this out”. “There’s a drug we can give him, his blood pressure is high but his age is for him, but if it goes up any more we’ll not be able to give him it”. [They say] “You have to decide, we can’t recommend it but there’s 3 scenarios. The first scenario is he could make a full recovery. The second scenario is it could leave him the same way as he is now and the third scenario is that it can cause a blood clot which could be fatal and by the way [interviewee laughs] you’ve got seven minutes”. (FamC02)*

Patient understanding of information, which is problematic in many healthcare encounters, is even more problematic in hyperacute stroke where the site of damage may affect cognitive processing. Clinicians reported that many stroke patients have problems with attention, concentration and memory which make grasping and retaining complex information difficult. Moreover, in the emergency setting, the immediate shock and trauma of the stroke was understood, by clinicians, to impair the capacity of patients to assimilate information.

*The issues in terms of thrombolysis… It is something that has happened, a stroke, has happened very quickly in somebody who often was perfectly well until the moment that happened. So they’re in shock. So they are in shock. The stroke itself may have affected how people think and communicate, and are often not able, not able, for a number of reasons, to either communicate for themselves or to take in the information that’s being given to them. (DocA02)*

*You’re never quite sure whether they’re in a state of shock […] So even, if you don’t have the issues of dysphasia [it] is still a question of whether the person can really engage given the severity of the circumstances. They’re suddenly yanked into hospital having been blue lighted and people say, ‘You’re seriously ill, we’ve got to give you this treatment which may prevent you from becoming very disabled’, how on Earth do you take all that on board? (DocC01)*

A stroke is sudden, unexpected and potentially life changing; a “shock”, reportedly, even for those patients at high risk or who had had a previous stroke. Stroke can manifest itself in ways that are physically debilitating and mentally incapacitating, and may be frightening to patients and their family. Effects may include impaired speech, immobility of limbs and exaggerated affect (emotionality). Furthermore, strokes vary in their intensity and severity. For patients the effects of shock and anxiety may have as much impact on their capacity for decision making as the direct effects of the stroke. Coming to terms rapidly with the stroke event itself was at the forefront of patient and family concerns: this necessarily shaped their engagement in decision making.

Some patients reported having difficulties in absorbing and understanding the information imparted by health professionals in the immediate hours following the event; that they were not in a position to ‘take in’ the information; and that they preferred to delegate information-processing to their family to help them to make the decision or to make it on their behalf. However some family members reported that their own capacity to make decisions was also compromised by the situation they found themselves in.

*Anything like that would have had to have been directed towards like my wife, the family rather than towards me because to be honest with you I was in no real fit state to be asked. (Patient(Pat)A05)*

*Pat: Well I mean she says they were talking to me but I can’t remember them talking to me about it.*

*Fam1: … well I thought I was taking it in completely but I was too worried about him.*

*Fam2: If whoever is with you, if they can’t take it in… You are trying to take it in but you are not fully realising what [the doctor] is saying to you. It is just like the big words stick out like ‘risk’ you know, ‘in 3 hours’ and all this, that and the other. And then you are watching whoever you are with. (Pat/FamC08)*

*I cannot tell you what [the doctor] said to be honest. I mean I, I was in such, I was in such a state of shock you must realise I was in complete shock at the time and I was trembling and my knees were trembling. I don’t know what I would have looked like to him but my knees were trembling and, and I, I am thinking to myself I can’t make a clear decision I must go and ask V [a family friend] Normally I would have made it myself but I was in, I was in terrible shock. (FamB07)*

Patients predominantly reported being unable to fully recall the immediate post-stroke events, or the information given to them at this time, but this was not a universal experience. Some family reported a general understanding of the information clinicians had given them.

*When he was explaining things he was explaining things in a way that a layman can understand. After a while he came through and again explained this treatment you could get saying that what it involved was a dramatic thinning of the blood to flush away any clots that might be there, em, but that it had to be done in a 3 hour time frame which the young doctor had already said (FamB04)*

#### Relationships and trust

From the patient and family perspective, a key issue of importance in their decision making was the care and support given by the health professionals who attended them, the support of their family and the sense of an organisation working effectively and efficiently. The social, emotional and decisional support of family and other trusted members of their social network were crucial (family friends: in one case a minister, in another a nurse).

As I say, just make sure you’ve got somebody with you. And if you go, like I did, into an accident and emergency by yourself make sure that your next of kin are hotly on the heels. To help you in case you have to make this decision. (PatC02)

Asked about their preference for engagement in decision making, patients and family reported a reliance on their clinicians, particularly their doctor. They valued their doctor’s expertise, whilst nonetheless expecting their own views to be taken into account.

*I always leave it to the doctor, he knows best what is going on, he knows best whether it is good for you or not. (PatA05)*

*He is the expert. He knows, I mean he’s, he’s the one with experience hopefully with experience and he’s the one that knows the ins and outs of it but also any, any reservations that I had I would like him to listen to what I had to say. (FamB07)*

Equally, clinicians reported a prevailing patient preference to let major clinical decisions rest with them – or at least be guided by them. For clinicians it was important to give hope and reassurance to the patient to allay anxiety.

*So I think the engagement is from a, a clinician’s point of view you’re trying to give hope, reassurance where you can in a context where actually the outcomes might be very bad. (DocC02)*

Reliance on clinical expertise in decision making did not, however, equate to a desire for paternalistic medicine. A clear expectation of respectful interactions with health care providers was evident in patient and family discussion of the relationship with a patient’s clinician. Trust was built on a frank interchanges which didn’t patronise: “he said ‘I need lots of questions answered quickly’ and he explained”.

*I-I just feel that as long as they are honest with you and explain everything very carefully, discuss it with you but don’t talk down to you, don’t patronise you. You see I didn’t have any problems because I just trusted. I think that was, I trusted Dr D because of his approach to us because when I went in he got me and he sat, knelt down in front of me, and he got both hands and I was shaking and he said “I need lots of questions answered quickly” and he explained (FamC04)*

Trust in their doctor and a need for reassurance was clearly important to patients and family. Their experience of stress and general confusion was reportedly helped by information about what was happening to them and around them, as well as by risk information about potential treatments. Largely this was through being informed by their health care team about the processes and procedures during their care trajectory.

### Knowledge Translation in the Emergency Setting

In the context of limited time for decision making about rtPA, there was little opportunity for the establishment and grounding of relationships, or for the reflection and deliberation typically expected of shared decision making [18], [19]. In this section, we discuss the strategies deployed by clinicians to ameliorate the seeming incommensurability of: lack of time, yet a need for reflection; blunted capacity for assimilating information, yet a desire to remain informed; and reliance on the expertise of clinicians yet an expectation that patient/family views are accounted for.

#### Face-to-face communication

With capacity and desire for information potentially blunted, patients and their family sought reassurance from healthcare professionals. This relationship became key in the decision making process. In accord with the importance of relationships and trust demonstrated above, patients’ and families’ emphasis was on information being given face-to-face from a trusted and knowledgeable professional. The prospect of having to assimilate written material, when patients and family were experiencing the immediate impact of stroke and very rapid decisions were required, was resisted by patients and family alike. Clinicians also preferred verbal presentation, reportedly because it allowed bespoke knowledge translation, i.e. the tailoring of information to individual patients.

*I couldn’t have read on the Wednesday night, I couldn’t have…erm it wouldn’t go in. If somebody’s talking to you, I can cope with that but not, not reading it, no. (PatA02)*

*I think if I was given a leaflet I would have been in too much of a state at the time to actually sit down and read it properly. Em, I think just maybe another professional person there present (FamA11)*

I mean some people like everything written down, other people… I mean there is not really that much you can write down that somebody doesn’t say. You know, “this is the opportunity, this drug’s a fairly good drug, it has got this bleed and that’s the two things that you have to balance up.” (FamC10)

*You know, according to those circumstances printed information is a possibility but it’s always better if it’s verbal by somebody. (DocB01)*

The effect on patients’ capacity to absorb complex information immediately post-stroke was widely reported by clinicians in the study, with clinicians on one site concluding that patient information sheets might not be appropriate for hyperacute stroke patients.

*Yeah, we have actually in my service discussed quite at length actually whether we have an information sheet or not. We felt it wouldn’t actually help in, in terms of the decision that people make. I think, the thought they would be trying to read this information sheet at the time when we’d rather they actually be listening to what we were saying and thinking about it, we just decided it really wouldn’t have a lot of additional value. (DocB01)*

On the other hand (i.e. the negative cases), some clinicians suggested value in written information and at least one clinician had drafted an information sheet for patients.

*I think probably the main bit of information you want to convey is that I’m being honest and open about the risks and the benefits and “here it is in black and white” and I actually give them a copy of it so that they can take it home, that they can later on say, ‘But you said this or that’, well you know, there it is that’s what I said to you. (DocC01)*

*Erm, so for certain family members that might be a useful time to say “Look, just a little leaflet, this is what a stroke is, you’ve got a blocked blood vessel, this drug dissolves it, therefore that minimises brain damage, these are the downsides”. Again, just explaining in writing what you’ve just tried to explain (Nurse(Nur)A05)*

Notably, the suggested provision of written information by Doctor C01 is not necessarily for use in the hyperacute phase, it is to be “taken home”. Moreover, there is an understanding that such information may not be appropriate for all, it is for “certain family members”. This view accorded with the small number of patients and family who would value written information but recognised that not everyone would.

*It would have been nice if I’d had a leaflet. I mean other people might not. Other people might have been put off by the printed word but I would not. I would have liked the information and my daughter would have done [so] as well if she had been there. Both of us would have been able to read and process that. Not everybody I think perhaps would have appreciated that. But somebody to have, sit down quietly beside them and explain the ins and outs. (FamC07)*

Negative cases were also evident among patient and family members. When asked if written information would be useful, some agreed that it would be in certain contexts. Demonstrating the power of the negative case to reinforce the prevailing view, they used this caveat to affirm the importance of face-to-face communication.

Int: [Would it] have been helpful to have something to read, or to watch, or…? I mean obviously you did have someone that quietly sat down and explained it to you, and that seemed to have come across as being…

Fam: Yes, that’s the most helpful. I find that yes, the leaflet is fine. You can be given it. You can read it when you get home. But it’s at the actual time when somebody sits and says to you this is the way it is, that’s what sticks in your mind most. (FamA01)

Some patients, family and clinicians identified the potential value of prior knowledge i.e. before the acute stroke event. In the absence of time to absorb new information, patients identified that the understandings needed to make decisions about rtPA were best known before a stroke; that is, that a general awareness of rtPA and stroke was needed, especially for those at higher risk.

*Int: Is there anything else that you think might be important in helping people in making a decision about thrombolysis.*

*Pat: I think just the fact that people should be informed beforehand you know, people who are more vulnerable to having a stroke possibly having this kind of information available to them so that should anything really happen then they have got that. Then their partners or whoever a GP or a doctor or whatever would know this and they could make an informed decision then, you know what I mean? (PatA05)*

Asked to think of other potential support to help assimilate information and make decisions in the hyperacute phase, patients and family returned to a relational form of support: a person to help translate, support and reassure them in the process. The potential for this form of support was recognised by clinicians too.

Yeah, it was pretty daunting. He was coming and saying this [is the decision that needs to be made], you know. If there was somebody who like would sort of be in between me and the doctor. Maybe a nurse or someone just to be relaying the information and making sure that everybody is understanding. Being there for me sort of thing, giving us encouragement. Saying “this is recommended treatment” or whatever. It’s just hearing it from somebody else I think sort of strengthens your belief that it is a good thing to do rather than just you and the doctor (FamA11)

#### Shaping decisions

Clinicians reported shaping what they saw as the ‘right decision’ for the patient. This was viewed positively by many patients, who found attempts by clinicians to portray information and choices from a neutral position as “unhelpful”. Patients valued doctors expressing confidence in their advice, and in avoiding giving a sense of uncertainty or delegating the decision to the patient; seen below in one case where the clinician’s approach had reportedly not been one of shaping the decision. In another example, when questioned about being “led” towards a decision, the interviewee resisted the negative implications of the question by positioning the doctor’s approach as “informing and involving”.

*I don’t think, to be truthful I don’t think we should’ve had to make the decision, cos, you go to the hospital to make you better not for you to make a decision. I think the way they should’ve put [it] was “there is this drug available, it can have serious side effects”, they could’ve told us exactly the same thing but putting it on the end was “we advise you to take it” and then we could have the consultant’s view instead of somebody who has got no idea about medicine (FamC02)*

*Int: Did you feel more that you were in their hands, or I’m sort of wondering whether you felt that you were left to make the decision or did you feel that you were being led by the doctors really?*

*Fam: Led is not really the right word. I think the fact that they kept us involved and informed throughout the whole process, and that Dr P a couple of times came out of the area where she was being treated and sat beside us and told us the next stage made it much easier and gave us much more confidence in making that decision. (FamB04)*

Clinicians emphasised the importance of giving hope and reassurance to the patient to allay anxiety. Lowering of anxiety levels was understood by clinicians as enabling patients to be in a better frame of mind to take in information and thereby consider decision-making options. Reduced anxiety was also understood by clinicians as potentially alleviating high blood pressure, a key contraindication for thrombolysis.

*“This is what we propose to do, cause I think it’s in your best interest. There’s a risk of bleeding, are you happy for us to go ahead?” And that’s the conversation. So it’s … I try to be fairly sure and confident in what I say to patients cause I think uncertainty is unhelpful to patients at this time, so that’s my personal approach (DocC02)*

Clinicians generally understood that how they presented themselves, their manner and approach, was important to the understanding and acceptance of the information conveyed. The communication afforded by these more relational aspects of decision support could reportedly accommodate the effects of the stroke itself and serve to alleviate the anxiety caused by the shock of the stroke event.

#### Making time

We found, as in other contexts [12], [20], [21] that decision making was a process not an event: even within the constraints produced by the emergency setting. While the patient’s trajectory following stroke includes the potential for an rtPA decision, some patients come off this trajectory very early, for example if they have an absolute contraindication to rtPA or arrive too late for treatment. Though showing some variation in practice, clinicians broadly reported managing knowledge translation along the care trajectory in an incremental manner, which entailed the continual provision and build-up of information about possible treatment protocols (including rtPA) in light of the developing knowledge of the patient’s clinical state. Timing of information provision about rtPA varied. Some clinicians reported early discussion of rtPA, others delayed discussion until CT scan results were available. Anxiety about unnecessarily raising patient and family expectations provided the rationale for delaying discussion.

*I start, I try to start general, I try to start general and broad and to see whether people are taking that information, taking that information in. I then go into more detailed information but adjust my language and detail according to how people are responding and you know their ability to take information at that time and their educational level. (DocA02)*

*I mean, it’s not just said once either, it’s all the time through. By the time we’re waiting on all the different investigations, you know, test results coming back and different things, getting all the information, all the time. It’s not just said once, it’s said a few times different ways and things. Do you know what I mean? So it’s not just going in and saying ‘Right, here you are you’ve got a choice, are you going to go with this, that and the other’. It’s a building up of it, if you like. (NurB06)*

*That is my style, to try and spread it out so that some of the things you say can sink in because I don’t think that it’s fair to have a pressured discussion at any stage during the process. I think one needs to plant some of the ideas and let them have a chance to think about it as they go, get wheeled down in the lift and all that. They also need to develop a confidence in the professionalism and just that the people looking after them know what they’re doing. And the sooner you start sharing some of the information with them, they start assessing what’s happening against what you’ve told them. And I think that helps them to build up confidence or on the other hand they might remain more cynical which is fair enough. At least you’ve armed them with some of the information. (DocC01)*

Though knowledge translation was constrained by the limited time available, this limitation necessitated the judicious use of that time. Adopting a strategy of consciously building up information content and complexity allowed for better assimilation of that information. Another product of this approach was that it could also function to build trust and confidence at a stage when there was much uncertainty for the patient and their family. The communication of information was a context sensitive and relational activity for patients, their family and clinicians.

*To be honest I think the important thing is the discussion that we have with the patients and the relatives. And that they are given time, limited though it is. But time to reflect on what we have said and what we have discussed and what our recommendation is and what the risks are. And given some time to try and analyse that and come up with whether it is right for them to go ahead or not (NurC05)*

#### Tailoring communication

Clinicians orientated towards risks of rtPA not as a set of ‘fixed’ facts to be communicated, rather, knowledge translation was tailored; risks were assessed and communicated in light of how this applied to the particular patient and their situation.

*I mean if there was somebody who had had a stroke, was not affected cognitively or speech or speech-wise and was able to fully engage and discuss things and was asking questions and driving, you know, driving the conversation and finding out what they want and the way they want it then I would respond. Then I would be flexible and respond to that. On the other hand if you have got somebody who, you know, there are some people who you can’t even start to have a conversation with. In that case you’d be with their family. But…a lot of people’s understanding of even what a stroke is, how it can affect them and what is likely to happen…. has to be, that’s the starting point to explain the consequences. And most people I think their starting point is there. (DocA02)*

There was evidence of considerable energy and thought being put into appropriate ways to present the risks and benefits, but also of struggling with some of the challenges of doing so in the context of the emergency setting and individual clinicians’ experience of delivering rtPA and risk communication. There was, therefore, considerable variation in the methods of risk communication. Clinicians varied knowledge translation between patients and sometimes with the same patient. Clinicians use mixed modes of information provision about rtPA, in part as a result of trying to convey the ‘necessary’ information to the patient. Using more than one mode of communication was felt likely to have more chance of succeeding. In attempting to meet the understandable desire on patients’ part for the information to be in lay terms, clinicians engaged in the creative use of language, forming similes and analogies in an attempt to convey meaning.

*I also talk in the same sort of language that I do with MIs. Which is, “there is a blockage in one of your arteries we can blast that by making your blood more leaky. Obviously by making your blood more leaky we risk making it leaky where we don’t want it to be leaky and it can leak out of where it is not meant to leak out of. It can leak into your gut, it can leak out into your brain. If it leaks into your brain that can cause a worse stroke or it can kill you” and you put in those sorts of terms. Then tell them about the risk of haemorrhage in terms of symptomatic bleeding, which is between three and five percent. But I usually explain that in terms of a one in twenty risk rather than [percentages] because people don’t tend to understand percentages […] so if you say it’s a it’s a one in twenty [chance], betting on horses and things like that, so they understand that a bit better [chuckle]. If you say it’s a twenty to one chance of having a haemorrhage that’s more comprehensible to most people. But usually, put it both ways. (DocC08)*

Concerned about potential inconsistency following an audit of risk information used by colleagues, clinicians in one site had prepared standardised risk information to facilitate knowledge translation, albeit, “tailored” to the particular situation and “needs” patient.

*So between ourselves, between the consultants who are doing it, we have got standard information that we give and tailor according to the needs of the patient. But we’re all quoting the same risks. (DocA02)*

### The Focus of Information

Despite the clear coherence between the needs of patients and family and of clinicians’ practices of knowledge translation, there appeared to be some disjuncture between what patients reported they wanted to *hear about* and what clinicians reported *discussing with them*. Patients reported a primary concern with their prognosis and likely outcomes, in particular emphasising social outcomes. The information and outcomes of interest reportedly required by patients and their family were, therefore, largely social in nature and predominantly related to ‘getting back to normal’ and the consequences for themselves and their family should this not be so.

*Well it [information] would be [about] my own health and that you know would I be capable of carrying on as I was before and doing all the things I did. Or would I have to depend on others I’d hate to be a nuisance or something for my daughter or anything you know. I’d want to really get over this. (PatA07)*

While it was clear that clinicians recognised the social and emotional context of decision making in their information sharing practice, they predominantly oriented to risk communication related to rtPA treatment and its physiological effects. When asked about information they would give to patients about treatment, clinicians largely talked of information in terms of the risk and benefits of treatment options. Though one clinician framed information in terms of social outcomes, these were restricted to physiological and practical outcomes in contrast to impacts upon psychological and emotional wellbeing or social interaction.

*Information to give to patients [I would] relate before administration of rtPA. So they have had a significant stroke caused by blocked artery in the brain. There may be some recovery but it’s hard to say how much at the moment, things could get worse. It’s not guaranteed to work. For [every] 10 patients we treat, one will have a good recovery. There’s also a risk of a new problem with this treatment. One in 30 chance of a serious complication of bleeding in the brain, this may be even fatal. (DocA01)*

Well I tend to focus on the things that you think would be the most important that anybody would want in their daily life really. You sort of say “well for the type of stroke they’ve had, um, the chances of them walking again is, you know, probably less than 50%” and “they’ve got a, um, an over 50% chance of going to a nursing home because of the actual support they would require at home” because you even build in things like “well, you know, do they live alone?” and you know already that if they’ve had a severe stroke, arm and leg aren’t working, can’t see on that side and they live alone in a house with stairs, the chances of *them getting* back to that home are very, very small. (DocB01)

While patients/family and clinicians considered both risk and social/contextual information, they each predominantly focused on one form above the other: patients/family – illness trajectory information; clinicians, especially doctors – risk communication. These different orientations to information reflect the differing objectives and responsibilities of patients/family and clinicians at this point in the care trajectory: respectively, to get better and to communicate the risks and benefits of treatment, in the context of decision making and consent, for the patient under their care.

## Discussion

Time constraints and the impact of the stroke itself shaped the practicable form, content and manner of knowledge translation to support decision making in the hyperacute setting. The need to make decisions quickly about rtPA meant there was little time for reflection. Cognitive impairments in patients and the shock of the event increased anxiety for both patient and family leading to problems assimilating information during this intensified decision making period. Patients often wanted to delegate decision making to family but family members’ capacity was also compromised. Patients sought social and emotional support from family and other trusted members of their social network.

Decision making about rtPA was heavily reliant upon health professionals’, especially doctors’, expertise. Patients and family nonetheless expected their views to be respected, they expected to be informed and they decried paternalistic or patronising communication. Decision making about rtPA in hyperacute stroke therefore presented three conundrums for patients, family and clinicians. First, how to allow time for reflection in a severely time-limited setting. Second, how to facilitate knowledge translation regarding important treatment risks and benefits when patient and family capacity is blunted by the effects and shock of stroke. Third, how to ensure patient and family views are taken into account where the situation produces reliance on the expertise of clinicians.

The strategies adopted to meet these challenges were fourfold: face to face communication; shaping decisions; incremental provision of information; and, communication tailored to the individual patient. First, with most patients and family finding written information difficult to assimilate in the hyperacute setting, the preferred mode of interaction was verbal and *face-to-face*. This relational form of interaction was understood to engender trust and allay anxiety, and was sought from health professionals, family and others in the patient’s social network. Second, clinicians reported *shaping decisions*. This was understood as an expression of confidence that offered hope and reassurance to patients and their family experiencing the shock of the stroke event and helped alleviate anxiety. Neutral presentations of information and treatment options promoted uncertainty and contributed to anxiety. Third, in the context of limited time, clinicians used a strategy of ‘drip feeding’ information to *make* time. Continual *incremental provision of information,* as appropriate to the patient’s emerging diagnosis, created small moments of time for reflection. Fourth, clinicians *tailored information* to the particular patient and family situation. Their use of language was creative and accounted for social and emotional contexts. Information was presented in a variety of ways and often more than once. In these ways clinicians were able to build the complexity of information in key stages, whilst also building trust and confidence. Nonetheless, the predominant focus of information was different for patients/family and clinicians, with the former focused on prognosis and likely outcomes of the stroke and the latter on risks and benefits of treatment. Thus, decision making and information sharing about rtPA in hyperacute stroke were necessarily relational and situated practices. That is, communication practices were shaped by the circumstances and context of decision making about stroke in the emergency setting, but also by the social and personal contexts, the emotional responses of patients and their families, and the shock and anxiety produced by the stroke event.

*Relational decision making* formed a primary mechanism for knowledge translation to support decision making and engagement of patients and family in decision making. Paradoxically, relational approaches to decision making produced practices of decision support that at face-value appeared contrary to prevailing views about patient autonomy. Respect for autonomy is commonly understood as the cornerstone of good ethical practice in contemporary health care with engagement in decision making the key strategy for preserving a patient’s autonomy [22]. Conventional conceptualisations understand patient autonomy as the performance of rational choice from a range of alternatives by independent autonomous individuals. In this context the neutral provision of information is presented as a goal of good risk communication and decision making. In this study the presentations by clinicians *shaped* decisions. While in the conventional view this could be seen as *jeopardising* patient autonomy, in this study this approach was viewed positively by many patients who found attempts by clinicians to portray information and choices from a neutral position as ‘unhelpful’. Patients and family valued doctors expressing confidence in their advice, and in avoiding giving a sense of uncertainty or delegating the decision to the patient.

We are not the first to note the incongruity of conventional approaches to autonomy. Others argue the primacy of autonomy in bioethics produces an imperative to choose which may itself be coercive [8], [23], [24]; emphasises rational decisions which fail to incorporate an individual’s values for given alternatives [25]; neglects other important aspects of autonomy such as self identity, self evaluation and capabilities for autonomy [26]; may limit the scope for action [27]; or, may poorly reflect actual decision making which may occur in circumstances of high emotion, accompanied by pain, discomfort, anxiety and concern for others [25]. Nor are we the first to demonstrate the importance of relational aspects of decision making. Ruiz-Moral argues convincingly that effectively engaging patients in decision making owes more to communicative efforts to achieve understanding, build trust and rapport rather than to an extensive discussion of possibilities or their prioritization [28]. Understandings of *relational autonomy* [*29*] may be more helpful in thinking about decision making. Supportive relationships may better facilitate autonomy than the provision of opportunities for independent decision making assumed by conventional understandings of autonomy [27]. Lown et al [30] have shown, albeit in a hypothetical setting, how patients, their families and clinicians acting together in a relational way was a necessary component of building trust and enabling the deeper enquiry needed to share concerns that influenced decision making, describing the relationship as a mutual responsibility, a partnership. In a study of decisions to proceed with allopathic stem cell transplantation, Forsyth et al [31] demonstrate the relational character and context of decision making. They show the interrelated impacts of physician expert opinions with the social, familial and community roles and interactions of the patient; that decision making was conducted in a ‘crowded room’. Certainly it was the interdependence of patients, their family and their health care team, in our study, that characterised descriptions of decision making and knowledge translation to support this decision making. We argue that the relational aspects of clinician interactions with patients and their families were key autonomy *promoting* practices. Our study is novel in demonstrating empirically how a relational approach to autonomy, by promoting a form of autonomy that accounts for and embraces the relational context of a patient’s decision-making, may thereby operate to enhance respect for patients despite an apparent threat to patient autonomy, as it is conventionally understood.

Alongside a relational approach to knowledge translation and decision making, a key mode of decision support was *situationally-sensitive* provision of information. While knowledge translation had both commonalities and variations, it was an on-going activity tailored to the social, emotional and clinical needs of patients and their families. Moreover, clinicians attempted to ameliorate the challenges of limited time by predominantly delivering information in a phased fashion. Clinicians were sensitive to the patient and family preferences produced by the setting: resistance to written information and preference for relational (face-to-face) risk communication. They customised their knowledge translation through creative uses of language and contextualising information content. Decision-making and risk communication in the hyperacute setting, as reported, thus demonstrated a degree of variation. Notwithstanding the benefits of personalised approaches to care and decision making variation in knowledge translation practices potentially produces challenges for risk communication.

### Strengths and Limitations

Qualitative research uniquely allows in depth examination of phenomena that are complex, value-laden and shaped by human interaction[32]–[34]. That qualitative methods enable access to the subjective experiences of those involved in the phenomena provides a perspective that is not available by more rigid pre-defined methods[32]–[34]. It is *only* through this in-depth exploratory and interpretative assessment of the perspectives of those directly involved in the activities of decision making in the emergency stroke setting that we are able to derive conclusions about what *is* important and therefore what support for decision making is actually needed. What we cannot determine from this study is the effect(s) of a *relationally* or *situationally-sensitive* approach in practice. The research method employed here, semi-structured qualitative interviews, produced accounts of what participants report as having occurred or not. This is not the same as the researchers witnessing the reported events and we can therefore present only reports of practice, not observations of the practice itself. This is an accepted limitation of this research method. Furthermore, questions asked of participants may produce apparent absences in their accounts; not because questions are biased but rather because respondents interpret them in relation to their relative experience of the phenomenon. These potential absences raise three notes for caution in our interpretation that necessarily shape our conclusions and recommendations. Further research that quantified the factors identified here may assist understanding of the characteristics of patients, family members and clinicians, and their associated preferences, to thereby better target decision support to population sub-groups.

First, while we have clearly demonstrated that provision of written information was resisted by most patients and family in the hyperacute phase, what is not apparent is whether other forms of prepared information might be desirable: respondents cannot describe modes of information provision with which they are unfamiliar. Moreover, patient and family resistance to written information was not resistance to knowledge translation *per se,* but rather to formal (largely written) information provision in the specific setting of emergency stroke. Nonetheless, this leads us to conclude that knowledge translation in the hyperacute setting should not comprise the more common forms of patient decision aid (eg. leaflets) which may be cognitively challenging and time consuming. Further research in this area to evaluate preferences and effectiveness with a range of alternatives is warranted.

Second, the different focus on desired information content by patients/family and clinicians, and the apparent emphasis by clinicians on facts rather than feelings, may in part be an artefact of the interview process. Clinicians were asked for information required for treatment decisions and, in the context of expectations about risk communication for evidence-based decision making, this is precisely what they offered in their accounts. More likely, however, this divergent emphasis represents *real* differences that reflect the differing objectives and responsibilities of patients/family and clinicians. For clinicians this responsibility must necessarily include attention to the advice and guidance of bodies such as GMC and NICE, hence they may be hyper-attuned to presenting the risks and benefits of treatment. Nonetheless, these different, though cognate, objectives and responsibilities comprise necessary components of the knowledge translation/risk communication process. The balance of ‘fact’ and ‘feeling’ needs careful consideration in developing decision support for patients and their families in the emergency setting.

Third, variation in information giving practices, whilst enabling care that is individualised and tailored to the specific needs of the patient, may introduce inaccuracies in the translation of evidence about risks and benefits. We have little evidence for the actual impact (positive or negative) of this variation and greater analytic understanding of their practical usage is required; especially as there appeared to be no formal training provided in practical risk communication.

### Conclusion

Standard rationalist approaches to knowledge translation and decision making risk leaving out the human element. Here we present a strong case to support existing clinical efforts to attend to the human element of knowledge translation/decision making; i.e. that it is contextual, carried out between people (relational) and made more difficult by the exigencies of the emergency setting.

The principal responses by clinicians to the challenges of decision making about rtPA in hyperacute stroke were *relational* decision support and *situationally-sensitive* knowledge translation. Into the future, these will undoubtedly form a crucial foundation for the effective implementation of personalised or precision medicine. But there is already evidence for reorienting the care pathway for hyperacute stroke to deliberatively accommodate the situational and relational components of knowledge translation and decision making to include three potential components of decision support. 1) Strengthen relational decision support practices as part of decision making training, including legitimating and acknowledging current good practice. 2) Provide risk communication and decision support materials for clinicians to support personalised communication of risks and benefits to relevant patient sub-groups (e.g. by age, sex, severity). 3) Provide opportunities for risk communication and decision support skills development for clinicians. Further research would better inform the strategies suggested here and may offer alternative means of supporting patients, families and staff in the decision making process.
